# Cognitive rehabilitation among long COVID patients using vibratory and auditory treatment (VAT) is linked to BDNF

**DOI:** 10.3389/fcogn.2025.1692578

**Published:** 2025-11-20

**Authors:** Abdullah Mosabbir, Jed A. Meltzer, Arkady Uryash, Erika L. Beroncal, Ana C. Andreazza, Lee Bartel

**Affiliations:** 1 Rotman Research Institute, Baycrest Academy for Research and Education, Toronto, ON, Canada; 2 Faculty of Music, St. George Campus, University of Toronto, Toronto, ON, Canada; 3 Department of Psychology, University of Toronto, Toronto, ON, Canada; 4 Department of Speech-language Pathology, University of Toronto, Toronto, ON, Canada; 5 Mount Sinai Medical Center, Miami Beach, Florida, FL, United States; 6 Department of Pharmacology and Toxicology, University of Toronto, Toronto, ON, Canada; 7 Centre for Addiction and Mental Health, Toronto, ON, Canada

**Keywords:** COVID, Long COVID, vibroacoustic, sound, cognition

## Abstract

Cognitive dysfunction occurs in around 40% of long COVID (LC) patients, and in many cases appears second only to fatigue in prevalence. Vibratory and auditory treatment (VAT) within the gamma range has demonstrated improvements in symptoms associated with cognition and fatigue. In this open-label pilot study, we tested the effects of VAT on measures of cognition and fatigue in LC. Twenty patients were randomly divided into a treatment and a control group. Symptoms were monitored remotely through mobile apps and in-person visits before and after the treatment period. The treatment group received a device generating 40 Hz of VAT to take home and use every day from Monday to Friday for 4 weeks (i.e., 20 sessions over 28 days), whereas the control group did not use any device but followed the same data collection procedures. This study found that after 4 weeks of VAT, participants with LC exhibited increased performance in selective attention and response inhibition, an increased amount of circulating brain-derived neurotrophic factor (BDNF), and a reduced resting heart rate. We propose that VAT may be a useful rehabilitative tool for LC as well as other targeted populations that seek improvements in cognition or general health but are compromised immunologically or physically.

## Introduction

It is increasingly being understood that COVID-19 has consequences beyond the respiratory system, leading to lingering health problems (Al-Aly et al., 2021). Such long-term issues have been described using varied terminology and descriptions but are now commonly referred to as either long COVID (LC), post-COVID-19 syndrome, or post-acute sequalae SARS-CoV-2. The National Institute for Health and Care Excellence (NICE) guidelines describes LC as “Signs or symptoms that develop during or after infection consistent with COVID-19, continue for more than 12 weeks and are not explained by an alternative diagnosis” (National Institute for Health Care Excellence, 2024). The primary features of LC include fatigue, dysautonomia (or postural orthostatic tachycardia syndrome), post-exertional malaise, and cognitive difficulties that are colloquially referred to as “brain fog” (Al-Aly and Rosen, 2024). Cognitive dysfunction occurs in around 40% of individuals with LC, and in many cases appears second only to fatigue in prevalence (Guo et al., 2022; Whitaker-Hardin et al., 2025). One study investigated over 800,000 adults from a larger community sample of more than 3 million individuals in the Real-time Assessment of Community Transmission (REACT) study of SARS-CoV-2 transmission in England (Hampshire et al., 2021). They found that those who had survived COVID-19, including those no longer reporting symptoms, exhibited significant cognitive deficits compared to controls when controlling for age, gender, education level, income, racial-ethnic group, pre-existing medical disorders, tiredness, depression, and anxiety.

The mechanisms of LC symptoms are still unclear, although preliminary evidence suggests that persistent neural inflammation plays a significant role in the development of cognitive deficits. Studies across brain-based disorders have demonstrated a convincing relationship between inflammatory markers and cognitive decline (Singh-Manoux et al., 2014), especially with chronic viral infections (Alford and Vera, 2018; Hoare et al., 1999; Rubin et al., 2018; Solinas et al., 2015; Weinstein et al., 2019). Postmortem brain data from COVID-19 victims indicate neural inflammation as one of the core features of SARS-CoV-2 exposure (Klein et al., 2021). The mechanisms behind neuronal damage caused by inflammation [which have been described extensively (Widmann and Heneka, 2014)], whereby systemic inflammatory cytokines may increase the permeability of the blood–brain barrier, allows toxic mediators into the brain and leads to the degeneration of neurons (Adams et al., 2019; Chen et al., 2020; Mehta et al., 2020). In COVID-19 patients requiring intensive care, it was reported that they had higher blood plasma levels of several pro-inflammatory cytokines (Huang et al., 2020; Tay et al., 2020). This aligns with other findings of COVID-19 survivors having poor cognitive test scores that are associated with specific inflammatory markers (Mazza et al., 2021).

Studies of vibratory and auditory treatment (VAT) within the gamma range (30–70 Hz, but especially 40 Hz) have demonstrated improvements in symptoms associated with cognition and fatigue (Bartel and Mosabbir, 2021). A recent review highlighted the use of multisensory stimulation therapy for the treatment of cognitive symptoms in many animal models of Alzheimer's disease. Specifically, they highlighted improvements in cognitive performance as well as physiological markers for 40 Hz sound, vibration, flickering light stimulation, or a combination thereof (Chen et al., 2025; Iaccarino et al., 2016; Martorell et al., 2019). VAT may also have a cardiovascular effect related to the release of nitric oxide (NO) by endothelial cells (Uryash and Adams, 2017a,b). The nitric oxide effect is known to upregulate brain-derived neurotrophic factor (BDNF) and glial-derived neurotrophic factor (GDNF), thereby contributing a neuroprotective effect (prior to damaging events) and a neurotherapeutic effect (following damaging events). Adams et al. (2014) showed that aerobic exercise significantly increases blood flow to the skeletal and heart muscles but not to the brain, while VAT increases blood flow (via BDNF and GDNF) to the brain and the heart but not to the muscles (Adams et al., 2014). They concluded that pulsed stimulation of the endothelium can be a non-invasive strategy for neuroprotection and neurotherapy. In this study, we hypothesize that VAT can be a possible treatment for LC patients who are experiencing cognitive impairment and fatigue, and the mechanism by which this occurs may be linked to the cardiovascular mechanism of NO in response to such stimulation. This mechanism may result in measurable changes in physiology (such as heart rate), inflammatory markers (BDNF and GDNF), and an overall improvement in cognition demonstrated by cognitive test performance. In this open-label study, we tested the effects of VAT on cognitive and physiological symptoms of patients with LC experiencing cognitive impairment and fatigue. Twenty patients were randomly allocated into one of two groups, a treatment group and a control group, and symptoms were monitored remotely through smartphone or tablet apps, as well as in-person visits before and after the treatment period. The treatment group received a VAT device generating 40 Hz of auditory and vibrotactile stimulation to take home and use every day from Monday to Friday for 4 weeks (i.e., 20 sessions over 28 days), whereas the control group did not use any device but followed the same data collection procedures. We hypothesize that 40 Hz VAT stimulation may improve cognitive ability in LC patients through the pulsed stimulation of the endothelium, via the release of cytokines or neurotrophins that circulate in the blood.

## Methods

### Study design

This is an open-label pilot study containing a group of LC patients self-reported with cognitive difficulties. The study is a collaboration between the University of Toronto, Canada, Baycrest Health Sciences Center, Toronto, Canada, and University College London, UK. The protocol was approved by Baycrest Health Sciences (REB 21-11) and the University of Toronto (REB 40907), where the research recruitment and procedures and labs were completed, and expedited ethics approval was obtained from all other institutions. Written informed consent was obtained from all participants in accordance with the Declaration of Helsinki. VAT has also been approved by the FDA for safety and increasing blood circulation, decreased pain, and increased mobility (Campbell, 2019). Once the experiment was complete, control participants were allowed to take the device home for 4 weeks to try the treatment regimen. This was for the ethical reason that we did not withhold any treatment from patients, but also ensured that control participants did not disengage to the point where they had to be removed from the study. Scores of cognition or physiology were generally not shared with participants, and they did not know their group allocation until the end of the baseline recording. This avoided any surprises or desire to “improve” or “reduce” a particular score. All experimenters used standardized scripts, and the NIH cognition toolbox was used via an iPad that showed all tests and text in a standardized manner. The NIH toolbox and western blots are validated measures. HR was measured using a phone app, which each participant was instructed on and taught to use repeatedly during the baseline visit until multiple recordings of similar values were achieved. They were instructed to relax during the use of the device at home at the same time of day, at night.

Twenty-two participants with self-reported cognitive symptoms post-COVID were recruited into this study, two of which were lost to follow-up. The participants were recruited from a LC support group on Facebook, and completed all research procedures at Baycrest Hospital. Inclusion criteria were COVID survival, at least 14 days since first symptoms appeared, 24 h without fever, and the ability to use and download digital applications on their mobile phone or tablet. Exclusion criteria included participation in another COVID-related trial, and exclusion related to pregnancy, hearing impairment, history of epilepsy/seizures, and other criteria as indicated in previous studies (Bartel and Mosabbir, 2021). Participants were randomized 1:1 using block randomization (block size = 4) implemented through a secure, central web-based randomization module. The randomization sequence was generated within the system and was inaccessible to the study team in advance. Allocation was revealed only after participant enrollment, ensuring allocation concealment.

### Participants

Basic demographic information was calculated from the NIH toolbox demographic questionnaire as well as short interview questions (Table 1). The average age of participants was 42.4 ± 12.3 years old, and most were diagnosed with COVID by their doctor or via a PCR test. The average number of days since their diagnosis date was 361 ± 264 days, with a minimum of 39 days and maximum of 791 days since diagnosis. Most participants contracted the original variant of COVID to their knowledge, and their symptoms persisted months after their diagnosis. Their primary complaints included the inability to focus, difficulty finding the right word to say, short-term memory problems, and fatigue. Other comorbidities were asked about through open-ended questions but were not specifically screened for. We screened for participants who did not have prior self-reported cognitive difficulties before the infection incident. Participants answer semi structured interviews regarding their symptoms, summarized in Table 2.

**Table 1:** Demographics for the entire Long-COVID sample.

**N = 22**	**Mean ±sd [*n* (%)]**	**Range (min – max)**
Age	42.4 ± 12.3	17 – 63
Sex	3M/19F	
**Type of diagnosis**		
PCR	8 (36)	
Doctor Diagnosis	5 (23)	
Rapid test	1 (4.5)	
Antibody test	1 (4.5)	
Pharmacy	1 (4.5)	
Unknown	6 (27)	
Days since COVID	361 ± 264	39 – 791
**Handedness**		
Right	20 (91)	
Left	2 (9)	
**Race**		
White	17 (77)	
Asian	3 (13.6)	
Other	2 (9)	
**Education**		
Professional degree (e.g. MD, DDS, JD, etc.)	2 (9)	High school – Professional degree
Master's degree	5 (22.7)	
Bachelor's degree	5 (22.7)	
One or more years of college, no degree	2 (9)	
Some college credit	2 (9)	
High school	2 (9)	
**Mother's education**		
Professional degree (e.g. MD, DDS, JD, etc.)	1 (4.5)	High school – Professional degree
Master's degree	3 (13.6)	
Bachelor's degree	4 (18)	
Associates degree	3 (13.6)	
One or more years of college, no degree	4 (18)	
High school	5 (22.7)	
Unknown	2 (9)	
**Primary complaint**		
Hard to focus	12 (55)	Cognitive or Fatigue
Word finding difficulty	10 (46)	
Short-term memory difficulty	8 (36)	
Fatigue	6 (27)	
Difficulty with daily tasks	4 (18)	
Headache	3 (13.6)	
All Others	15 (9)	

Out of 22 participants, 20 completed the study.

**Table 2:** Interview responses describing Long-COVID symptoms.

**Primary complaints**	**Other symptoms**
• Could not handle long conversations • Thinking made me tired and I would grasp at words that just would not come to me.	• Joint pain • Muscle pain • Fatigue • Shortness of breath • Occasional headaches • My penmanship actually got worse.
• I have trouble finding words that I know before • I mixed up sequence of words • My short term memory is almost non-existent.	• Chest tightness • Shortness of Breath • Sleep disorder • Fatigue • Cold feet • Cold fingers • Headaches • Burning eyes • Palpitation • Acid reflux • Muscle/joint pain • Tinnitus • Dizziness • Higher blood pressure • Running nose • Random tears • Double vision
• Initially I did not have any neurological symptoms. Those began to appear gradually a couple of months after I had COVID (I had other symptoms throughout that time). My brain fog symptoms are: • Difficulties with word finding • Mental slowness/trouble with information processing • Mental fatigue that comes on really quickly • Trouble concentrating • Difficulty with mental calculation • Memory problems	• Fatigue • Shortness of breath • Tinnitus • Tachycardia and lightheadedness when upright (Postural orthostatic tachycardia syndrome) • Post rational symptom exacerbation • Tremor (This has now gone away) • Frequent waking during the night • Exercise intolerance
• Headache • Hard to focus • Exhausted-Eyes hard to keep open or focus.	• Cough • Shortness of Breath • Racing heart • Swelling and vein issues in legs • Loss of smell and taste
• Inability to focus or concentrate • A break in the chain of what belongs to me (relational connections), particularly objects, skills • Lack of clarity • Easily irritated, moved to being reactive • Sleep affected- falling asleep, waking up • I would feel fuzzy (Head not clear) • Decreased capacity for abstraction and holding concepts.	• Tiredness, physical but cognitive as well • Easily affected by noises, temperature changes (easily feeling cold or hot) • Fatigue easily • Ear feeling blocked and heavy • Easily congested.
• Confusion • Cognitive dysfunction • Inability to focus • Reading and watching TV became difficult • Extreme fatigue • Dizziness • Shortness of breath exacerbated these symptoms.	• Chest pain • Shortness of breath • Radiating pain in left arm • Muscle aches • Dizziness and light headedness • Orthostatic intolerance • Extreme weakness in the arms • Headache • Numbness in left hand and arm • Low grade fever • Dry cough (intermittent) • Chills and flu like symptoms • Hives and rashes (went away after a few month) • Back pain • Post exertional malaise
• Inability to focus, recall information, understand what is being asked and unable to say the correct word for what I am talking about	• Fatigue • Loss of taste (completely) • Intermittent loss of smell • Body aches • Sore throat • Cough • Digestive issues
• Brain Fog • Forgetfulness • Forgetting words • Foggy • Difficulty focusing	• Breathing issues • High heart rate • Walking/dizziness • Fatigue • Blurring vision
• Difficulty focusing my attention • Difficulty with memory-especially short term memory.	• Fatigue- my most prominent symptom (Especially post exertional fatigue after physical/mental activity) • Sleep does not feel restorative. It takes a longer time to recover
• Headaches • Pressure • Confusion • Slowness • Lack of coordination • Sensitivity to light and sound • Slurred speech • Inability to read and write • Vision disturbances	• Shortness of breath • Rapid and irregular heartbeat • Nerve pain • Cough • Brain fog • Headaches with auras • Indigestion • Sleep disturbances • Body aches and pains
• Trouble concentrating • Intermittent loss of cognitive ability • Difficulty remembering certain words or names of people I know • Difficulty reading and remembering the information in a book • Very forgetful- confused- difficulty with calendars.	• Trouble sleeping • Dizzy spells • ALT level increase • Vivid nightmares • Blackouts • Trembling hands • Balance issues • Difficulty breathing w/exertion • Rash • Fatigue • Difficulty going upstairs • Anxiety • Heavy feeling limbs • Hair loss • Loss of taste + smell + temp swings
• Appeared after getting the vaccine (2nd dose), Aug 2021	• Fatigue • Brain fog
• Short term memory issues • Using the wrong words • Feeling like I am in a haze • Putting things away in the wrong place (e.g., finding the cheese in the living room laptop drawer) • Difficulty concentrating (e.g., hard to follow directions to new board game)	• Shortness of breath • Post exertion pain and malaise (inability to exercise) • Chest pain in the center of chest (near constant in first ear, now presents after exercise) • Difficulty regulating body temperature • Menstrual cycle changes • Hair loss (first year, since resolved) • Numbness in hands, feet (first 18–24 months, seems to have resolved now) • Brain fog • Significant fatigue
• Word finding difficulty, difficulty with written fluency as compared to baseline • Short term memory issues • Challenges with focus and executive functions (organization, doing steps logically) • Procession issues- slow to process, unable to process multiple things at the one time	• Fatigue • Heart rate elevation with standing
• Memory gap • Word finding difficulty • Inability to multitask when I used to be able to • Lack of focus • Concentration when I used to be able to • Memory lapses • Cognitive difficulty especially as the day wears on • Screen intolerance	• Extreme fatigue • Muscle aches • Blurry vision • Inflammation • COVID headaches • Unexplained weight gain • Incontinent diarrhea • Flare ups of other chronic pain (fibromyalgia) symptoms • Brain fog • Low energy • Exercise Intolerance • Loss of appetite • Food aversion • Nausea • Vertigo • Dizziness
• Forgetfulness/confusion • Difficulty in processing information • Attention span, concentration issues • Mental/cognitive fatigue • Post-exertional malaise/fatigue after activities requiring concentration/focus	• Neuropathic pain • Post exertional fatigue/malaise • General fatigue • Dysautonomia • Chronic fatigue symptom and fibromyalgia symptom
• Could not complete a sentence sometimes • Cannot find a word, yet can describe • Stop and rethink where I am going, when out	• Neurologia in legs + arms • Numbing left side of the body • Extreme fatigue • Headaches • Sore eyes, ankles, wrists • Breathing and heart rate changes • Mobility decrease

### Treatment schedule

The VAT consisted of 30 min of vibrotactile and auditory stimulation at 40 Hz, with the auditory source file including a 160 Hz sine wave amplitude modulated at 40 Hz. The sound track was streamed from Sound Cloud phone application through a supplied Sound Oasis VTS1000 device. The Sound Oasis VTS1000 device is a portable device that was supplied to the participant for use at home. The procedure included two study visits to Baycrest Hospital before and after the 4 weeks of VAT. During their study visits, they completed interview questions, NIH toolbox cognitive tests, a blood draw, and a short EEG session. They were instructed to use the device during any part of the day, every day from Monday to Friday, for 4 weeks at home. To maintain compliance, we checked Sound Cloud track usage analytics to see if the sound track was being played 5 times every week. HR measurements taken in the phone app contained timestamps for when they were taken. These were viewed during the experiment as a measure of compliance. The treatment and control groups had compliance rates of 91.9% and 88.7%, respectively. We believe this is an acceptable level of compliance and believe there is not a big difference between the groups in terms of engagement in the research. Before and after 30 min of vibration, they were to use a heart rate monitor application on their phone. Once every week, they were instructed to use a phone application that would test their cognitive ability through a set of games called EQ brain performance (available at https://highmark.tech/).

Venous blood samples were sent to a research lab located at the University of Toronto for storage and preparation, and then analyzed at Mt. Sinai medical center in Miami. In this pilot study, cytokine and neurotrophin levels were quantified primarily by western blot. Band intensities were normalized and quantified by densitometry (local background-corrected band intensity per area), yielding values in arbitrary units that reflect relative protein levels. ELISA assays were also performed for cross-validation, but western blot data were used for the main outcome analysis. Venous blood samples were collected in EDTA-tubes and processed at the University of Toronto. Using a Ficoll density-centrifugation protocol, plasma and peripheral blood mononuclear cells were extracted and stored in −80 °C until all samples were collected. The primary outcome of this study was cognitive test score changes and neurotrophin levels, whereas all others were secondary.

### NIH toolbox cognitive tests

The following cognitive tests from the NIH toolbox cognition battery were used:

- Dimension Change Card Sorting. This test uses color and shape matching to assess cognitive flexibility (Zelazo et al., 2014).
- Flanker Inhibitory Control and Attention task. This measures the ability participants to sustain attention and distinguish between concordant and discordant cues when presented with flanking stimuli (Zelazo et al., 2014).
- List Sorting Working Memory Task. This measures working memory—participants are presented with a sequence of pictures and verbal descriptions in a specific order (Tulsky et al., 2014).
- Oral Reading Recognition. This tests the ability of participants to pronounce specific words (Gershon et al., 2013).
- Picture Vocabulary Test. This tests participants' vocabulary when they listen to a word and select a picture that best matches the word's meaning (Gershon et al., 2013).
- Pattern Comparison Test. This test measures processing speed by asking participants to distinguish whether two visual stimuli are the same (Carlozzi et al., 2015).
- Picture Sequence Memory Test. This measures episodic memory by asking participants to recall the order in which they listened to and watched a sequence of pictures and events (Dikmen et al., 2014).

Two scores were then calculated based on the results of these tests:

- Fluid composite score. This score represents the capacity for new learning and information processing in novel situations. It comprises a combination of the results of the Dimensional Change Card Sorting, Flanker Inhibitory Control and Attention, Picture Sequence Memory (Form A), List Sorting Working Memory, and Pattern Comparison tests.
- Crystallized composite score. This score represents cognition based on experience such as the store of verbal knowledge and skills, abilities that are more influenced by education and cultural exposure, and relatively resistant to decline with advanced age and neurological disease. This score comprises the Picture Vocabulary and Oral Reading Recognition tests (Akshoomoff et al., 2013).

### Data processing and statistical analysis

Statistical analysis was conducted using R software. Independent variables were the group (treatment, control) and session (before treatment period, after treatment period). Dependent variables included cognitive scores, cytokine/neurotrophin levels, and heart rate values. A total of 17 tests were conducted, which includes repeated measures ANOVA, *t-*tests, and Pearson's correlation. Multiple test correction was performed using the Benjamini–Hochberg false discovery rate, including all tests. Reported results include the *p-*values adjusted for multiple tests and effect sizes. Supplemental Table 1 is also available for original *p-*value, adjusted *p-*value, effect size, and the 95% confidence interval for each test.

## Results

### Baseline cognitive scores

**Figure 1:**
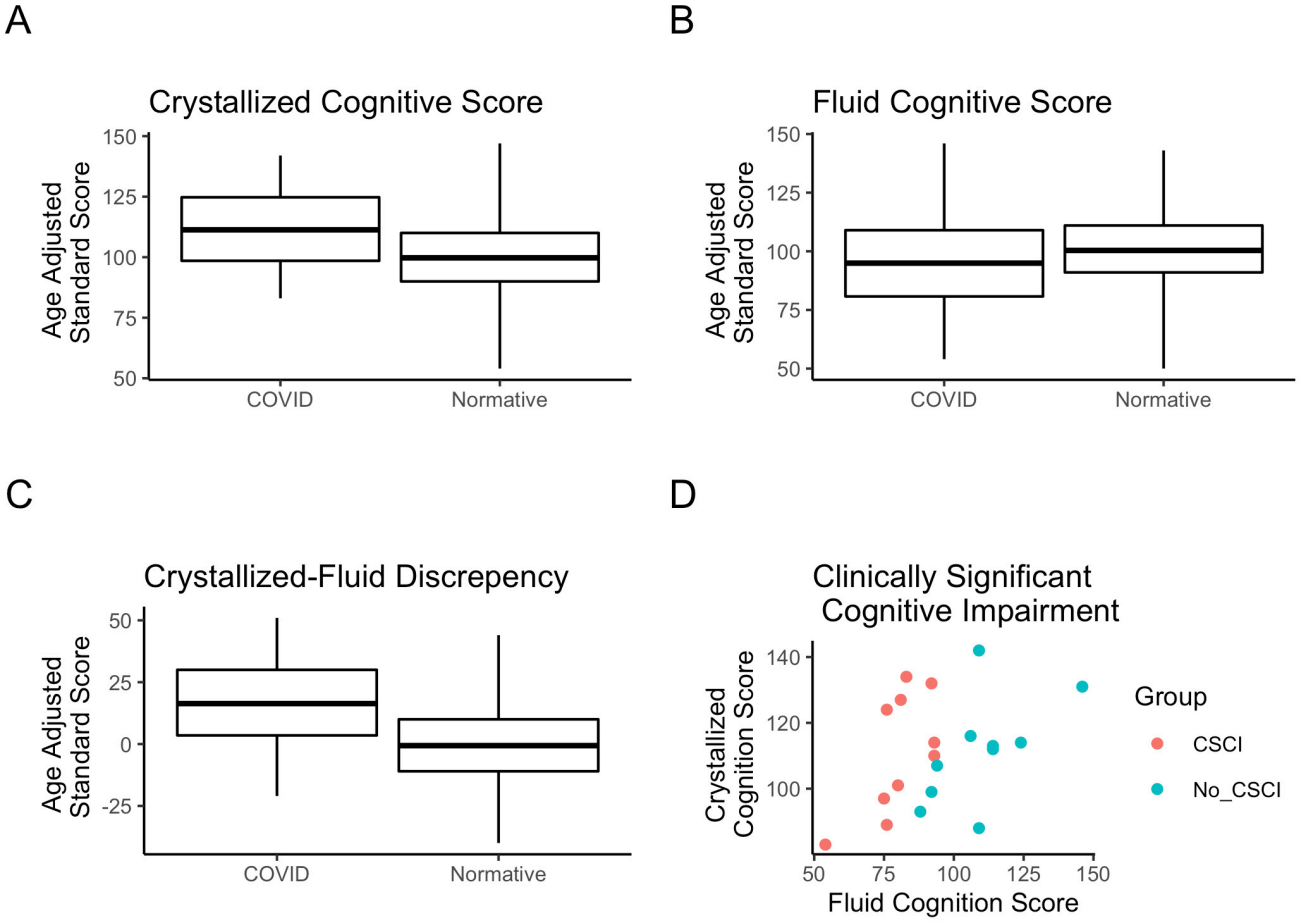
Normative cognitive scores compared to the LC sample. **(A)** Age-adjusted standard scores for crystallized cognition for the normative sample of 730 healthy adults compared to the LC sample. **(B)** Age-adjusted standard scores for fluid cognition in the normative vs. sample data. **(C)** Crystallized-to-fluid score discrepancy in the normative vs. sample data. **(D)** Scatter plot indicating the score for crystallized vs. fluid scores for each of the 20 participants who completed the study. Colors indicate which participants were considered as having clinically significant cognitive impairment, which is defined by a previous study (Jett et al., 2023). Box plot values depict the mean as the horizontal line, box edges at the 25th and 75th percentiles, and vertical lines indicating minimum and maximum values available.

The first assessment done was to compare baseline cognition to normative data for the NIH toolbox cognition scores. Normative values were obtained from a previously published study (*n* = 730; *M* = 47.4 years old, SD = 17.6, range: 18–85; 64.4% women; 63.1% White) (Iverson et al., 2023) Crystallized cognition represents a person's intelligence from acquired information, and is thus resilient to neurotrauma or early neurodegeneration. Compared to normative data, the sample data had a significantly greater average score based on a two-tailed *Z*-test (*Z* = 3.456, *M*_COVID_ = 111.3, *M*_norm_ = 99.74, *p*_adjusted_ = 0.003, Cohen's *d* (cd) = 0.770), indicating that the sample population had a greater level of acquired intelligence than a normal healthy population (Figure 1A). Fluid cognition, in contrast, represents an individual's ability to process and integrate information, act, and solve problems. This is sensitive to cognitive dysfunction and aging (Tucker-Drob, 2022; Tulsky et al., 2017). When comparing normative values to sample data, the comparison did not reveal a significant difference after adjusting for multiple comparisons (*Z* = −1.627, *M*_COVID_ = 94.95, *M*_norm_ = 100.37, *p*_adjusted_ = 0.1, cd = 0.26; Figure 1B). Crystallized scores and fluid scores, however, are considered to be correlated, such that the discrepancy between crystallized-to-fluid scores can be used as a measure of cognitive deficit or learning disabilities (Iverson et al., 2023). Crystallized scores stay relatively resistant to decline with advanced age and neurological disease, whereas fluid scores decline. Discrepancy scores derived from the NIH toolbox can be used as a within-person interpretive approach for detecting cognitive decline from pre-injury or pre-disease. The greater the discrepancy score, the greater the level of cognitive impairment. Discrepancy scores of normative data compared to sample data indicate a highly significant difference, where the LC sample has a greater score than the healthy population (*Z* = 5.07, *M*_COVID_ = 16.35, *M*_norm_ = −0.63, *p* = 0.0000004; Figure 1C). Another measure of cognitive impairment from crystallized and fluid scores is illustrated in Jett et al. (2023), where clinically significant cognitive impairment is defined as the presence of two or more fluid subtest scores falling 1.0–1.5 SD below the participant's crystallized composite score (Jett et al., 2023). Based on this definition, it was found that 10 (50%) of the LC participants who completed the study were considered impaired at the start of the study (Figure 1D). The allocation of participants to the treatment or control group was randomized, and we found exactly five considered impaired in each group.

### Post-treatment cognitive scores

**Figure 2:**
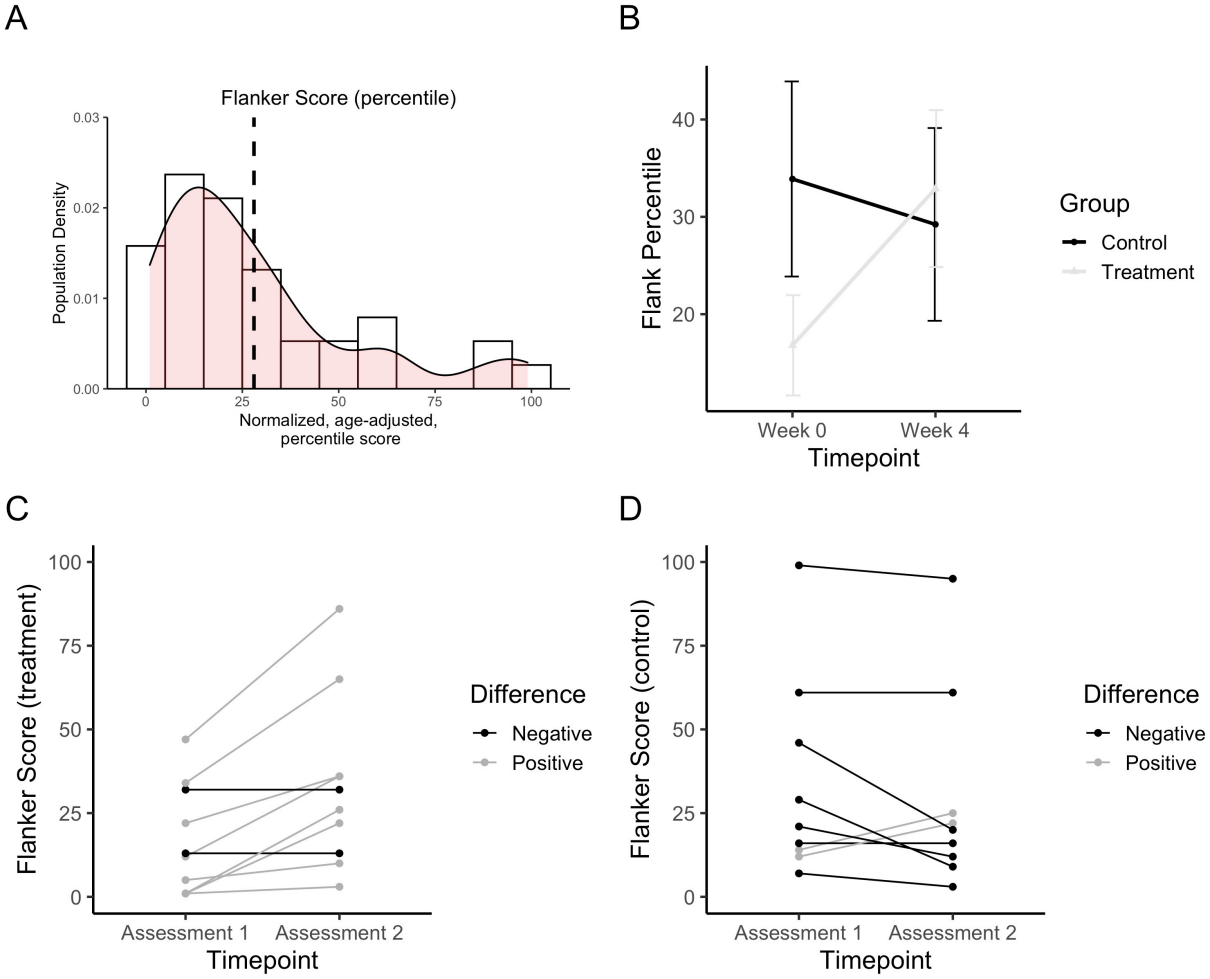
Fluid subtest (flanker test) scores after 4 weeks of VAT. **(A)** Density plot depicting the proportion of the population at specific percentiles scores compared to normative data. Scores represent normalized, age-adjusted percentile scores. **(B)** Flanker test scores before and after VAT for the treatment group vs. the control group. The dashed black line represents the mean score for the group. **(C)** Flanker test scores before and after VAT for each individual in the treatment group. **(D)** Flanker test scores before and after VAT for each individual in the control group.

After 4 weeks of VAT, participants were tested again for cognitive ability as well as plasma cytokine/neurotrophin levels. Measures were collected before and after the treatment period for the treatment and control groups. The flanker test, a fluid cognition subtest measuring attention and executive function, was found to be statistically significant in a repeated measures ANOVA assessment for a group-by-session interaction effect [*F*_(1, 1)_ = 11.69, *p*_adjusted_ = 0.014, partial eta squared (pe^2^) = 0.41; Figure 2B]. Baseline measures of the flanker test indicated that the majority of individuals had a percentile score below the 50th percentile (Figure 2A). Individual flanker scores show that 8 (80%) members of the treatment group improved in the flanker test after VAT (Figure 2C), whereas only 2 (20%) members of the control group improved (Figure 2D).

### Post-treatment neurotrophin levels

**Figure 3:**
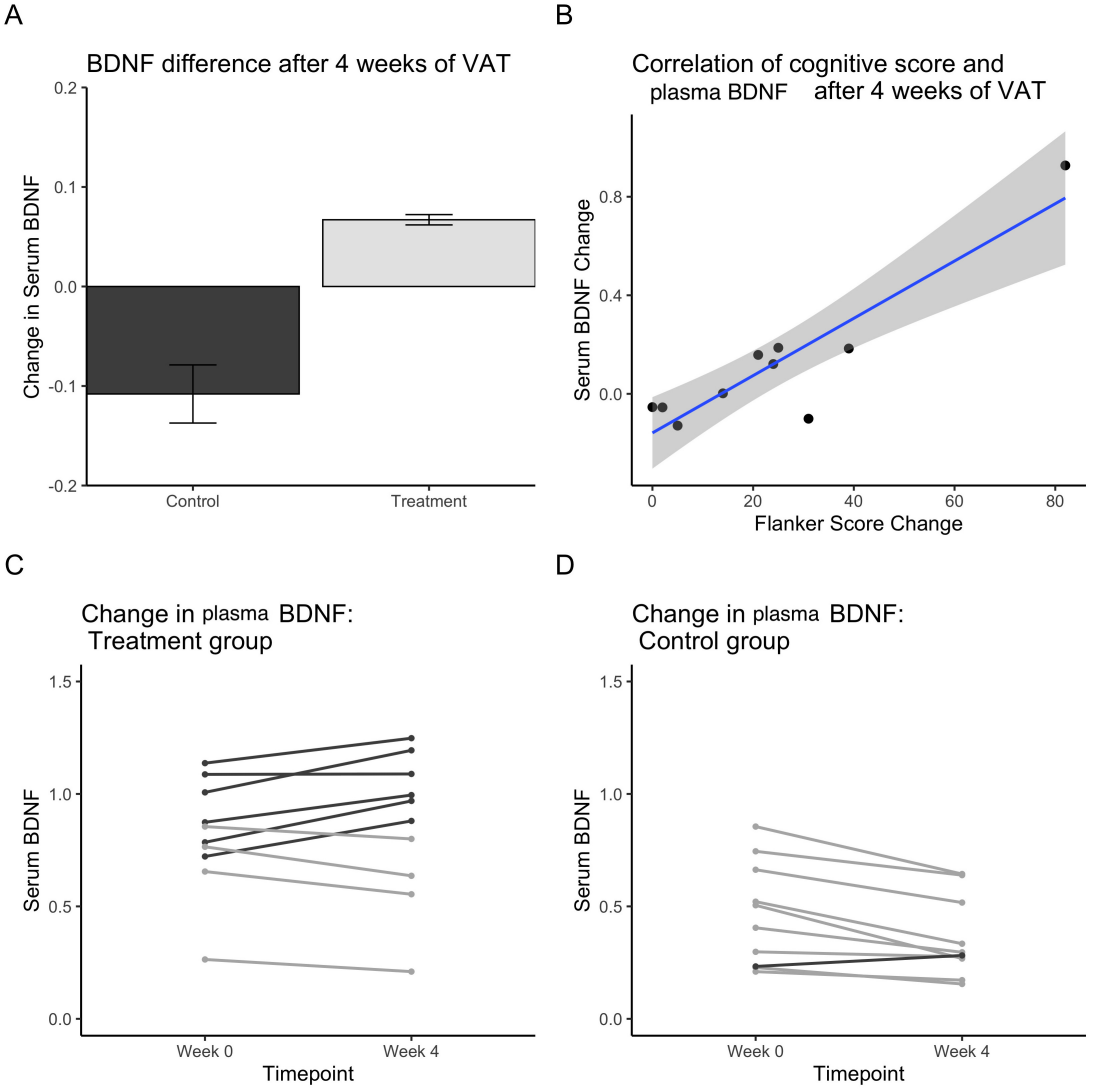
Plasma BDNF values from western blot data after 4 weeks of VAT. **(A)** Plasma BDNF change after 4 weeks of VAT for the treatment group vs. the control group. **(B)** Line plot depicting the correlation between the change in plasma BDNF scores and the change in flanker percentile score. **(C)** The change in plasma BDNF for each individual in the treatment group. **(D)** The change in plasma BDNF for each individual in the control group.

Plasma neurotrophin levels were collected before and after the treatment period for the treatment and control groups. Change in plasma BDNF, a neurotrophin important for neuron survival and growth, was found to be statistically significant in a repeated measures ANOVA assessment for a group-by-session interaction effect [*F*_(1, 1)_ = 9.76, *p*_adjusted_ = 0.0314, pe^2^ = 0.22; Figure 3A]. Six factors known to be neuroprotective were of interest in this study (IL6, IL10, TNF-a, IFN-y, GDNF, and BDNF). Linear regression between the change in flanker test and change in plasma BDNF value was found to be statistically significant (*F* = 37.79, $R_{\text{adjusted}}^{2}$ = 0.825, *p*_adjusted_ = 0.003; Figure 3B). When examining the change in BDNF for each individual, it was found that 6 (60%) of participants from the treatment group and 1 (10%) of the control group increased in BDNF after 4 weeks of VAT Figures 3C, D).

### Physiological markers after treatment

**Figure 4:**
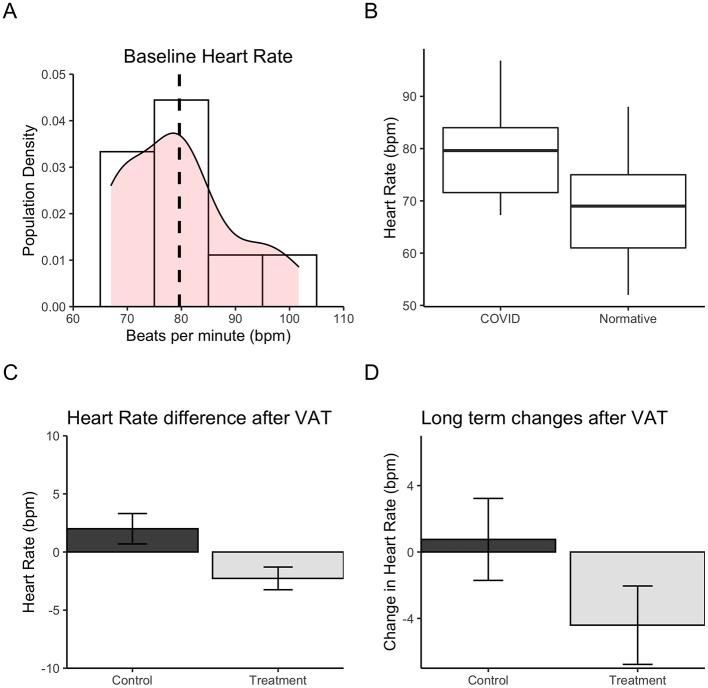
Heart rate change after 4 weeks of VAT. **(A)** Density plot depicting the distribution of heart rate scores (beats per minute) for the LC sample. Mean sample heart rate is indicated by the dashed vertical line. **(B)** Heart rate (bpm) difference between the LC sample and normative heart rate data for healthy adult population in Canada. The horizontal line indicates mean, box edges represent 25th and 75th quartiles, and vertical lines indicate minimum and maximum values. **(C)** Heart rate change immediately after each 30-minute VAT session for the treatment group vs. control group. **(D)** Heart rate change after 4 weeks of VAT for the treatment group vs. control group. Long-term change is calculated by the difference in pre-vibration heart rate in the first 3 days vs. the last 3 days of the 4-week treatment period.

Heart rate (HR) and heart rate variability were measured before and after VAT for both groups. Baseline measures indicated that the average HR was 80 beats per minute (bpm) with the majority ranging between 70 and 85 bpm (Figure 4A). Comparing these values to the normative HR, we see that there is a statistically significant difference (*Z* = 4.576, *M*_COVID_ = 79.62, *M*_norm_ = 69.0, *p*_adjusted_ = 0.003, cd = 1.01), whereby the LC sample had a greater HR than the healthy population at baseline (Figure 4B). Short-term change in HR was calculated by subtracting the HR before and immediately after VAT in each session, averaged for 4 weeks of daily use. There was a significant change in HR after short-term VAT (*t* = −2.61, d*f* = 14.811, *p*_adjusted_ = 0.04; Figure 4C). Participants were instructed to be seated for 30 minutes in a sedentary setting and to relax during the procedure. Reading a book or watching a movie were suggested as actions that were allowed during the treatment. This was to control the activity of each participant and reduce variation in short-term HR measurements. Although 30 min of sedentary activity could decrease HR, we were interested in seeing if there was a difference between the groups. HR change was also calculated for long-term VAT use as an average of pre-VAT use within the first 3 days compared to pre-VAT use within the last 3 days. Pre-VAT HR values were unaffected by short-term VAT use but the change in pre-VAT HR over days illustrates how the HR may change over long-term VAT usage. Long-term VAT produced HR changes that were statistically significant in a repeated measures ANOVA test for a group-by-session interaction [*F*_(1, 1)_ = 46.92, *p*_adjusted_ = 0.027, pe^2^ = 0.365]. HR significantly decreased over time in the treatment group compared to the control group (Figures 4C, D).

## Discussion

Twenty patients who self-reported cognitive impairment after infection of COVID-19 were recruited in this study, which took place in 2022–23. Since self-reported views of cognitive impairment are typically inconsistent compared to tested measures of impairment, we aimed to test whether our sample demonstrated these impairments using the NIH toolbox cognitive battery. We tested crystallized and fluid cognitive scores at baseline compared to normative values. Over 16,000 subjects completed the NIH toolbox cognition battery norming project, and their scores were adjusted for age, education, sex, race, and ethnicity (Casaletto et al., 2015). The LC sample had a greater crystallized score compared to normative results, whereas their fluid scores were below norms. This suggests that this sample does indeed have some form of impairment. Fluid to crystallized ratios are suggested to be close to 1:1 (Iverson et al., 2023), and so the baseline crystallized scores being above norms may suggest that the magnitude of fluid score decrease could be large. Using another definition of cognitive impairment via NIH toolbox scores, namely having two or more fluid subtest scores falling 1.0–1.5 SD below crystallized, we found that 50% of the patients were classified as having cognitive impairment. Usually, the relationship between self-reported vs. tested cognitive impairment is inconsistent, with one study stating a conversion rate of 24.5% for mild cognitive impairment (Cuevas et al., 2022; Wion et al., 2022). Therefore, a rate of 50% for this sample is considered high, and our confidence for cognitive impairment being a serious issue for LC participants is strengthened by this result.

This study found that after 4 weeks of VAT, participants with LC experienced an increased performance in selective attention and response inhibition as measured by the flanker task, tapping abilities that are a common subject of complaint among those self-reporting with cognitive impairments. Improvement on the flanker task was found to correlate with an increased amount of circulating BDNF. One of the mechanisms of VAT is to produce an exercise-like effect on the skeletal muscles, whereby vibration stimulates the muscle stretch reflex, leading to multiple downstream health benefits (Bartel and Mosabbir, 2021). Sustained aerobic exercise has been shown to improve cognition, lower heart rate, and increase BDNF over time (Huang et al., 2023). Exercise factors (irisin, cathepsin B, CLU, and GPLD1) are released from skeletal muscles, the largest secretory organ of the body, leading to crosstalk with other body organs. Notably, these exercise factors can cross the blood–brain barrier and stimulate the release of neurotrophic factors such as BDNF (Chen et al., 2022). Another factor which may be involved is nitric oxide (NO), which is a signaling molecule that triggers a cascade of events that lead to smooth muscle relaxation and vasodilation (Zhao et al., 2015). One of the downstream effects of this is to upregulate BDNF, which serves as a switch for neural progenitor cells to transition from proliferation to differentiation. This relationship to BDNF forms a positive feedback loop, where BDNF can upregulate the expression of nitric oxide synthase in neurons, allowing them to produce more NO (Banoujaafar et al., 2016; Cheng et al., 2003). Another effect of NO, independent of BDNF, is its role in the immune response to viral infection, as it may inhibit viral replication, promoting viral clearance and the recovery of the host (Reiss and Komatsu, 1998). Compounds that increase the production of NO or mimic its effects have been shown to reduce the replication of SARS-CoV-2 specifically, and have been suggested as a potential therapeutic (Akerström et al., 2005; Akaberi et al., 2020). Further research is needed to clarify whether an increase in NO that results in the reduction of viral replication can be elicited directly from VAT.

Currently, the options for cognitive rehabilitation for LC in the literature are few, and include cognitive training, non-invasive brain stimulation, targeted pharmacological interventions, and exercise rehabilitation (Whitaker-Hardin et al., 2025). Although similarities are present, VAT is not equivalent to exercise. The results of exercise on BDNF may vary based on age or exercise intensity, as one study found that different intensities of exercise failed to increase BDNF (Murawska-Ciałowicz et al., 2021). In addition to that, VAT may offer some advantages that exercise does not. It is well known that exercise produces an increase in inflammation over the short term, which could inadvertently worsen conditions for those with a compromised immune system or those at risk of an aberrant immune response (Cerqueira et al., 2020). This could be of concern for those with LC, who often complain of post-exertional malaise. VAT however, has been associated with producing an anti-inflammatory response in the body, and thus may serve as a useful alternative rehabilitative tool for LC patients (Moreira-Marconi et al., 2022). This may extend to other patient populations that have a similar hurdle with regards to exercise, such as those with physical disabilities, limited mobility, chronic pain or fatigue, advanced age or frailty, or short-term conditions where rest is necessary (i.e., post-surgical recovery).

Another factor to explore in understanding the mechanism of the positive health effects of VAT would be in brain imaging measures, particularly those measuring rhythmic brain activity (EEG/MEG). An emerging hypothesis is that neural entrainment from pulsed stimuli such as VAT can synchronize neurons that maximally oscillate at those frequencies (Iaccarino et al., 2016; Lakatos et al., 2019). Vibratory stimuli at 20 Hz or 40 Hz may produce an increase in power in resonance frequencies in the short term, called a steady state response (Vakorin et al., 2018; Ross et al., 2013), or an increase in gamma range resting state power in the long term (Mosabbir et al., 2022; Sahu and Tseng, 2023). Furthermore, gamma power has been associated with increased attention and focus, and has been correlated positively with BDNF (Huang and Morozov, 2011; Hiramoto et al., 2017; Jones et al., 2018). Therefore, future studies correlating the rhythmic brain activity in response to VAT and its relationship to cognitive function would contribute greatly to our understanding of the mechanism of VAT. A tertiary reason why this phenomenon is important to investigate is the hypothesis that multiple neurological conditions may be associated with abnormal rhythmic activity, and that it may be alleviated with a “reset” through pulsed stimuli at specific frequency patterns. This may prove a useful avenue to explore conditions that are difficult to understand, such as chronic pain or fibromyalgia.

Future research will require greater efforts to explore the correlations found in this pilot study. Studies that are randomized and controlled via placebo/sham while exploring more cytokines or neurotrophins will strengthen our understanding of the mechanistic link between BDNF, cognition, and VAT. The absence of sham and assessor blinding could introduce expectation bias, and therefore a more rigorous follow-up study is necessary to solidify any findings from this study. Specifically, an active control would be required that would receive an alternative task, an established standard treatment, or a placebo task. The main purpose of using an active control is to isolate the specific effect of the new intervention by excluding potential confounding variables like the placebo effect (improvement due to expectation), the Hawthorne effect (improvement due to attention), or the natural progression of time. Although we found a strong correlation between BDNF and attentional skills, we reserve the interpretation of any causation until these results can be independently replicated with more rigor. Future studies should also include neuroimaging of rhythmic brain activity such as electroencephalography or magnetoencephalography. Furthermore, there may be bias in self-reports of cognitive and physiological adherence, and so a rigorous cognitive test to identify and screen participants with cognitive impairment beforehand would also strengthen the results. Investigating other frequencies of stimulation of VAT also have merit. We chose 40 Hz because it is the most frequently tested frequency within the gamma range (30–80 Hz). Gamma range stimulation in vibroacoustic therapy is suggested to be involved with improving cognitive symptoms. We hypothesized that pulsed stimulation of the endothelium can stimulate the release of cytokines and neurotrophins that can improve cognitive symptoms in long COVID. It is not known whether a different band of stimulation frequency can produce a similar result, how these frequencies compare in the release of factors, and how sensitive a change in these frequencies may change physiological outcomes. Since this is a pilot study, it was more feasible for us to consider the most used frequency in the most reported frequency band. Although we found a strong correlation between BDNF and attentional skills, we reserve interpreting any causation until these results can be independently replicated with more rigor.

In summary, we found that 4 weeks of VAT at a stimulation frequency of 40 Hz was correlated with improved cognition and physiological markers in a sample of LC patients. This improvement was linked to an increase in plasma BDNF, suggesting a possible mechanism for VAT. We propose that VAT may be a useful rehabilitative tool for LC as well as other targeted populations that need benefits in cognition or general health but are compromised immunologically or physically.

## Data Availability

The raw data supporting the conclusions of this article will be made available by the authors, without undue reservation.
