# Supplementary material for: Cognitive rehabilitation among long COVID patients using vibratory and auditory treatment (VAT) is linked to BDNF
Source: Front Cognit. 2025 Nov 20;4:1692578. doi: 10.3389/fcogn.2025.1692578 (PMC13281226; doi:10.3389/fcogn.2025.1692578)
Supplement: Supplementary file 1 [file Data_Sheet_1.docx]

Supplemental Table 1: Statistics results for each comparison.

| Test Name | p-value | FDR adjusted p | Effect Size | 95% Confidence Interval |
| --- | --- | --- | --- | --- |
|  |  |  |  |  |
| Cognitive |  |  |  |  |
| Crystallized norm | 0.00054 | 0.00306 | 0.770 (d) | [0.324, 1.216] |
| Fluid norm | 0.05160 | 0.10965 | 0.260 (d) | [-0.71, 0.18] |
| Flanker | 0.00327 | 0.01389 | 0.410 (pe^2^) | [7.95, 33.6] |
| Card Sorting | 0.31900 | 0.47175 | 0.060 (pe^2^) | [-15, 43.6] |
| Picture Sequence | 0.15580 | 0.29428 | 0.110 (pe^2^) | [-50.3, 8.7] |
| List Sorting | 0.19800 | 0.33660 | 0.090 (pe^2^) | [-6.63, 29.8] |
| Pattern Comp | 0.51100 | 0.57913 | 0.020 (pe^2^) | [-15.2, 30.2] |
|  |  |  |  |  |
| Blood |  |  |  |  |
| IL6 | 0.94900 | 0.9490 | 0.0002 (pe^2^) | [-0.212, 0.225] |
| IL10 | 0.39800 | 0.4832 | 0.040 (pe^2^) | [-0.415, 0.174] |
| TNF-a | 0.86500 | 0.9190 | 0.002 (pe^2^) | [-0.383, 0.45] |
| IFN-y | 0.33300 | 0.4717 | 0.060 (pe^2^) | [-0.135, 0.376] |
| GDNF | 0.36300 | 0.4746 | 0.050 (pe^2^) | [-0.168, 0.0647] |
| BDNF | 0.01110 | 0.0314 | 0.220 (pe^2^) | [0.0163, 0.455] |
| BDNF vs. Fluid | 0.00028 | 0.0030 | 0.825 (R^2^) | [0.65, 0.98] |
|  |  |  |  |  |
| Physiological |  |  |  |  |
| HR normative | 0.00040 | 0.0030 | 1.010 (d) | [0.57, 1.45] |
| HR short term | 0.01950 | 0.04735 | 1.170 (d) | [-7.753, -0.791] |
| HR long term | 0.00790 | 0.02686 | 0.365 (pe^2^) | [0.08, 0.60] |

Tests completed include repeated measures anova, t-test, and pearson’s correlation. For repeated measures anova, the p-value represents the group by session interaction effect. The effect size for repeated measures anova test is partial eta squared (pe2), for t-test it is cohen’s d (d), and for correlation the R-squared (R^2^).

Supplemental Table 2: A CONSORT style schematic table.

| Recruitment Stage | Number of Participants |
| --- | --- |
| Assessed for eligibility | 50 |
| Randomized | 22 |
| Allocated to each group | 10 treatment; 12 control |
| Completed each study visit | 10 treatment; 10 control |
| Included in primary analysis | 10 treatment; 10 control |
